# Linking metabolic and contractile dysfunction in aged cardiac myocytes

**DOI:** 10.14814/phy2.13485

**Published:** 2017-10-30

**Authors:** Gregory P. Barton, Willem J. de Lange, John C. Ralphe, Judd Aiken, Gary Diffee

**Affiliations:** ^1^ Balke Biodynamics Laboratory Department of Kinesiology University of Wisconsin‐Madison Madison Wisconsin; ^2^ Department of Pediatrics University of Wisconsin‐Madison Madison Wisconsin; ^3^ Department of Agriculture, Food, and Nutritional Sciences University of Alberta Edmonton Alberta Canada

**Keywords:** Aging, cardiac myocytes, contractile function, mitochondrial function

## Abstract

Aging is associated with declining cardiac contractile function as well as changes in metabolism and mitochondrial function. The relationship between age‐related changes in cardiac metabolism and declining cardiac contractile function has not been determined. In order to define the role energetics play in changes in contractile function, we measured mitochondrial NADH, [NADH]_m_, during continuous contractions of isolated left ventricular myocytes from young (Y) and old (O) FBN rats. Second, we explored the role of metabolic disruption with rotenone and increased workload with isoproterenol (ISO) had on age‐related changes in myocytes shortening. Single, intact myocytes were stimulated for 10 min of continuous contraction at either 2 Hz or 4 Hz while being perfused with Ringer's solution. Properties of shortening (peak shortening and rate of shortening) were measured at the onset (T0) and after 10 min (T10) of continuous contraction, and the decline in shortening over time (T10/T0) was determined. Although young and old myocytes had similar contractile function under resting conditions, old myocytes demonstrated decrements in [NADH]_m_ during continuous stimulation, while young myocytes maintained constant [NADH]m over this time. In addition, old myocytes exhibited impaired contractile function to a workload (ISO) and metabolic (rotenone) stress compared to young myocytes. Taken together, these results demonstrated that old myocytes are susceptible to stress‐induced contractile dysfunction which may be related to altered cellular energetics.

## Introduction

Myocardial contractile function is diminished with age and contributes to cardiac morbidity and mortality in the elderly (Lakatta et al. 2001; Yang et al. 2005). A number of factors contribute to the pathogenesis of cardiac aging including action potential prolongation, altered myosin heavy chain isoform expression, mitochondrial defects and free radical accumulation, decreased cell number, increased fibrosis, and Ca^2+^ handling dysregulation (Anversa et al. 1990; Lakatta and Sollott 2002; Yang et al. 2005; Hacker et al. 2006; Preston et al. 2008). Recent evidence points to a role of altered cardiac energy metabolism in the onset and development of cardiac aging (Abu‐Erreish et al. 1977; McMillin et al. 1993; Schocke et al. 2003; Sample and Cleland 2006; Bhashyam et al. 2007), but the precise role of changes in cardiac metabolism in the aging heart has not been clearly delineated. Aging hearts have a demonstrated reduction in enzymes involved in both the tricarboxylic acid (TCA) cycle and the electron transport chain (ETC), and these changes are coupled to a reduction in oxygen consumption and ATP formation (Kumaran et al. 2005; Preston et al. 2008).

Age‐related changes in metabolic or mitochondrial function would theoretically have a negative impact on myocardial contractile performance in the aging heart, but this link has not been fully explored. The mammalian heart has a limited capacity to store chemical energy and yet is capable of increasing pump work threefold during periods of high demand (Balaban 2002; Carley et al. 2014). The rapid and dramatic capacity for work is achieved through an increase in flux through energy metabolism pathways, with minimal changes in the concentrations of ATP and ADP. Myocardial work is exquisitely coupled to ATP turnover (Balaban et al. 1986; Mootha et al. 1997). At high workloads, the ATP pool can turn over in as little as 2 sec (Mootha et al. 1997). ATP pool turnover is related to the mitochondrial respiratory rate, thus, modest changes in cardiac metabolic capacity may have a significant impact on contractile function. Changes in energy metabolism occur in parallel or precede contractile dysfunction in models of pressure‐overload hypertrophy (Pound et al. 2009; Doenst et al. 2010; Kolwicz et al. 2012). Furthermore, enhancing mitochondrial substrate metabolism, either pharmacologically or genetically, has been shown to mitigate the decline in contractile function in pressure‐overload hypertrophy (Pound et al. 2009; Kolwicz et al. 2012). However, the impact of altered energetics on contractile function in aging alone, without the presence of other pathologies, remains to be explored.

In this study, we used contractile performance of intact isolated myocytes to determine the relationship between contractile changes and metabolic changes. In order to focus specifically on contractile consequences of altered metabolic function, we studied contractile properties of cells during 10 min of continuous stimulation. We hypothesized that declines in cellular contraction over this stimulation period would be more significant in aged cardiomyocytes and reflective of age‐related metabolic changes occurring within the cell. We used NADH fluorescence as a measure of mitochondrial respiration, taking advantage of the linear relationship between mitochondrial respiration, ATP turnover, and NADH levels (Mootha et al. 1997). We found that cardiomyocytes from aged hearts had a significant decline in contractile function during conditions of increased stress compared to younger hearts, and that this decline was likely related to decreased mitochondrial respiratory function.

## Methods

### Animals

Six‐month‐old and 33‐month‐old male Fischer 344 x Brown Norway hybrid rats (FBN) were obtained from the National Institute on Aging colony at Harlan Industries (Indianapolis, IN). The FBN hybrid rat is a long‐lived strain with a median life span of 33 months and a maximum life span of 40 months. The FBN rat is considered a “healthy aging model” widely used and highly recommended for gerontological research. All rats were confined to standard size rodent cages and housed two rats per cage. Rats had access to food and water ad libitum and were acclimated to reverse daylight (12 h dark, 12 h light). Animal housing and handling was carried out under the guidelines of the University of Wisconsin‐Madison Institutional Animal Care and Use Committees and conducted in pathogen‐free facilities that are accredited by the American Association of Accreditation of Laboratory Animal Care.

### Cardiomyocyte isolation

Rats were euthanized using isoflurane/pheumothorax euthanasia. Hearts were rapidly removed from the rats and mounted onto a temperature‐controlled (37°C) Langendorff system (Fraticelli et al. 1989; Walker et al. 1993; Wahr et al. 2000). Hearts were perfused with Ringer's solution in mmol/L: 118 NaCl, 4.8 KCl, 1.2 NaH_2_PO_3_, 25 HEPES, 11 glucose, 1.2 MgCl_2_, 1 CaCl_2_, at pH 7.4, and gassed with 5% CO_2_–95% O_2_. After perfusing with Ca^2+^ Ringer's for 3 min, the hearts were digested with Ringer's (Ca^2+^ free) containing type II collagenase and hyaluronidase for 25 min. The digested heart was removed from the cannula, and the ventricles cut into smaller pieces and placed in a 37°C water bath for another 25 min of digestion in Ringer's (Ca^2+^ free) containing type II collagenase and hyaluronidase. Tissue pieces were gently triturated and the suspension centrifuged to pellet the cells. Extracellular Ca^2+^ was added incrementally back to 1 mmol/L by centrifugation in Ringer's containing first 200 *μ*mol/L Ca^2+^. Only rod‐shaped myocytes with clear sarcomere striations were chosen for contractile studies.

### Cell shortening and mitochondrial NADH measurement

Simultaneous cell shortening and NADH fluorescence measurements were performed at 37°C. Cells were placed into a Warner chamber and superfused with glucose Ringer's solution (2 mmol/L Ca^2+^) and stimulated at 4 Hz with platinum electrodes. Changes in cell length during shortening were acquired at time 0 (T0) and after 10 min (T10) of continuous contraction. The change in contractile properties over time was assessed by the arithmetic ratio of (T10/T0). Contractile properties were assessed using a SoftEdge MyoCam^®^ system (IonOptix Corp., Milton, MA, USA). The myocyte being studied was displayed on a computer monitor using an IonOptix MyoCam camera. An IonOptix SoftEdge software was used to capture changes in cell length during shortening and relengthening. Cell shortening was assessed using the following indices: peak shortening (distance shortened/diastolic cell length × 100), an indication of peak ventricular contractility. The maximal velocity of shortening (+dL/dt), calculated as the maximal slope derivative of the shortening phase and is an indication of maximal velocity of ventricular pressure increase.

### Mitochondrial NADH autofluorescence [NADH]_m_

**Figure 1:**
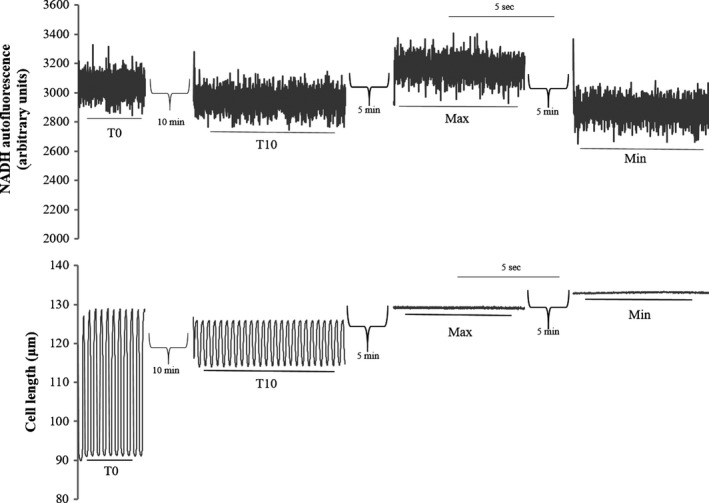
Representative trace of an old cardiac myocyte during simultaneous contractile and NADH measurements. T0 measurements occur after 30 sec of contraction in order to ensure steady‐state NADH tracings and T10 measurements occurred after 10 min of continuous stimulation. The “Max” and “Min” represent the maximum and minimum NADH fluorescence obtained using cyanide and FCCP, respectively, as described in Methods. The Max and Min measures were performed after the 10 min of continuous stimulation while the myocyte was quiescent. In this case, T0 and T10 NADH levels were 63% and 23% of max, respectively.

NADH autofluorescence was measured in cells in which contractile measurements were made by using methods similar to those described elsewhere (Eng et al. 1989; White and Wittenberg 1993; Griffiths et al. 1997, 1998; Brandes and Bers 2002; Heinzel et al. 2006; Jo et al. 2006). Briefly, cells were illuminated with 340 nm light through a Plan‐Flour 40× objective on an Eclipse Ti microscope (Nikon). Similarly, emitted light was passed through the same Plan‐Flour 40× objective, and 460 nm emission was measured using IonOptix hardware and software. Both contractile data and emission data were digitized at 250 Hz. To minimize photobleaching and the artifact due to cell contraction, the excitation light was applied for only 10 sec during contractile measurements at T0 and then again at T10. Since intracellular NADH cannot be calibrated in absolute terms, the intensity of NADH fluorescence was expressed as a percentage of full‐scale change in the fluorescence in the presence of 5 mmol/L NaCN to give maximum NADH fluorescence and then in the presence of 5 *μ*mol/L carbonyl cyanide 4–(trifluoromethoxy) phenylhydrazone (FCCP) to give minimum NADH fluorescence, respectively, at the end of each experiment (Jo et al. 2006). We used the following equation to calculate NADH levels: % NADH = 100 × (F – minimum; F)/(maximum F – minimum; F), where F is the fluorescence intensity measured. A representation of the simultaneous measurement of cell length changes and NADH fluorescence is shown in Figure 1.

In order to determine contractile consequences of decreased mitochondrial function, we measured single‐cell contractile properties in cells following administration of rotenone, a known inhibitor of mitochondrial oxidative phosphorylation. For these experiments, contractile properties of cardiac myocytes were assessed by visualizing the cell through a Hitachi camera, with the video image collected at 60 frames/second. Cell shortening was measured using edge detection software created in LabView (National Instruments, Austin, TX). Cells were placed in a Warner chamber mounted on a stage of an inverted microscope (Olympus, CK 40). After cardiac myocytes contracted initially (T0) in glucose Ringer's solution with 0.15% DMSO at 2 Hz, we rapidly perfused with a glucose Ringer's solution containing 1 *μ*mol/L rotenone in 0.15% DMSO using the MP Series Chamber Manifold (Warner Instruments, Inc.). Rotenone concentration was chosen and modified based on studies of rotenone effects in intact hearts (Lesnefsky et al. 2004). Cell shortening measurements were recorded (T10) after 10 min of continuous contraction in rotenone.

For isoproterenol exposure experiments, cells were superfused (1 mL/min at 37°C) with glucose Ringer's containing 100 nmol/L isoproterenol. Isoproterenol concentration was modified from previous experiments on cardiac myocytes (Guarnieri et al. 1980; Hayes et al. 1984; Jiang et al. 1993). Cells were allowed to incubate in the isoproterenol buffered solution for 5 min prior to the acquisition of cell shortening measurements. The cells were field stimulated at a frequency of 2 Hz and changes in cell length were measured at time 0 (T0) and after 5 min (T5) of contraction. Unlike the myocytes from young animals, many old myocytes were unable to last the entire continuous stimulation protocol, which was even more pronounced during the administration of rotenone or isoproterenol due to the development of arrhythmias or cessation of contraction, which was why we used 2 Hz stimulation in these experiments instead of 4 Hz.

**Figure 2:**
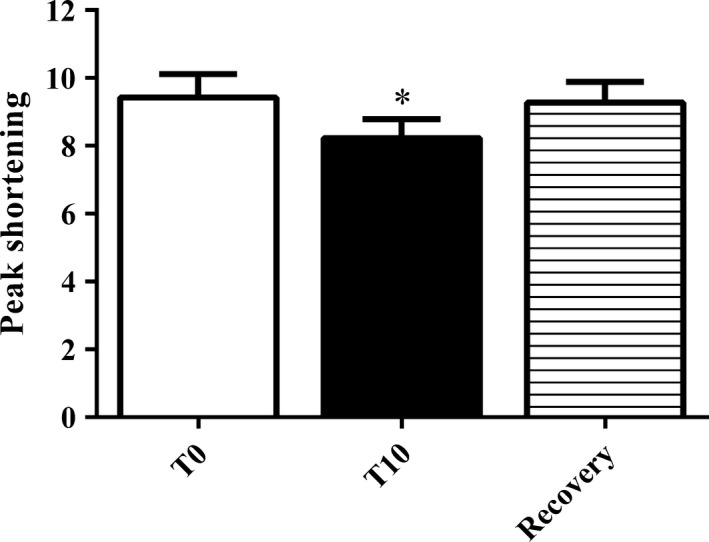
Effects of recovery period on peak shortening. After 10 min of continuous stimulation, peak shortening decreased by ~13% at T10 compared to T0. Following 10 min of quiescence, we restimulated the myocytes and measured peak shortening, and found that peak shortening recovered to ~99% of the value at T0. *n* = 18 myocytes. Values represent means ± SE. *P < 0.05 main effect of continuous stimulation.

Since myocyte contractile properties decline over time, we performed quality control experiments on old myocytes superfused (1 mL/min at 37°C) with glucose Ringer's, the results are shown in Figure 2. The cells were field stimulated at a frequency of 2 Hz and changes in cell length were measured at time 0 (T0) and after 10 min (T10) of contraction. After the T10 measurement, we turned off the stimulation and continued to perfuse the cells with Ringer's solution for 10 min of quiescence. After the 10 min of quiescence, we restimulated the cells and measured cell length changes after the recovery period.

### Statistical analysis

Two‐way ANOVA was used to determine differences for NADH measurements and contractile properties in the rotenone experiments. Post hoc analysis was performed when the two‐way ANOVA was significant using Tukey's LSD to determine between‐group differences. Between‐group means for the contractile properties in the baseline 4 Hz and for the isoproterenol experiments were analyzed by the Mann–Whitney test. Within‐group differences in contractile properties in the baseline 4 Hz was determined by the Wilcoxon matched pairs signed‐rank test. One‐way ANOVA was used to determine differences in the effect of recovery on myocyte contractile performance. All statistics were performed with (GraphPad Prism 6; GraphPad Software Inc., San Diego, CA). Significance was determined at *P* < 0.05. All values are represented as means ± SE.

## Results

### Contractile measurements at 4 Hz

Contractile properties for young and old cardiac myocytes are shown in Table 1. All contractile properties measured significantly declined over 10 min of continuous stimulation in both young and old myocytes (T0 vs. T10; *P* < 0.05), but the T0 versus T10 decline was not different between young and old myocytes.

**Table 1:** Contractile properties in young and old myocytes stimulated continuously for 10 min at 4 Hz

	Young	Old
Peak shortening		
T0	10.39 ± 0.51	11.11 ± 0.80
T10	6.59 ± 0.33^a^	7.04 ± 0.37^a^
T10/T0	0.66 ± 0.03	0.68 ± 0.04
+dL/dt (*μ*m/sec)		
T0	271 ± 18.16	264 ± 17.46
T10	157 ± 10.15^a^	165 ± 9.43^a^
T10/T0	0.62 ± 0.04	0.67 ± 0.05
Diastolic cell length (*μ*m)		
T0	111 ± 3.62	113 ± 3.51
T10	104 ± 3.95^a^	104 ± 4.02^a^
T10/T0	0.936 ± 0.01	0.916 ± 0.01

Values reflect means ± SE. T0 = initial values; T10 = values after 10 min of continuous stimulation; T10/T0 = ratio of the above values. *n* = 30 (young), *n* = 34 (old).

a Within‐group differences relative to T0, *P* < 0.05.

### Effect of recovery period on peak shortening

In order to determine that the decline in contractile properties over time is in fact due to temporary metabolic dysfunction as a result of the contractile activity, or a permanent decline in function due to the cell isolation procedure, we allowed a 10‐min recovery period following the 10‐min continuous stimulation and then restimulated the myocytes to measure function. Cell length changes were determined at T0, T10, and after 10 min of recovery (Fig. 2). Peak shortening significantly declined from T0 to T10 (T0: 9.4 ± 0.7 vs. T10: 8.2 ± 0.6). Peak shortening increased after 10 min of quiet rest (recovery: 9.3 ± 0.6), indicating that the decline in function is likely due to intracellular changes accumulating during the stimulation.

### Mitochondrial NADH fluorescence [NADH]_m_

**Figure 3:**
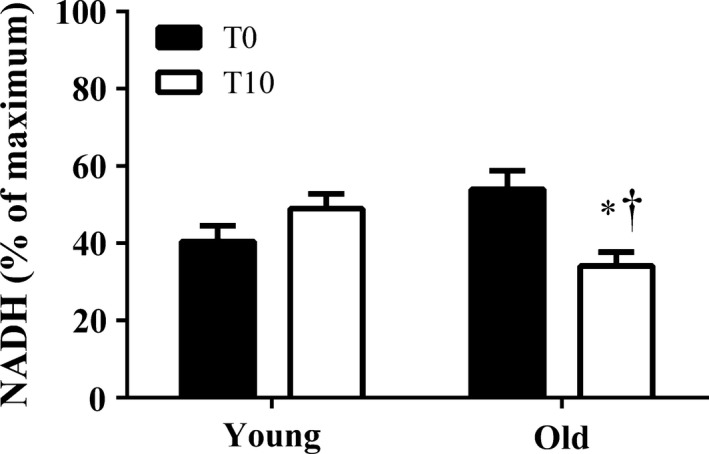
Single‐cell NADH concentrations at T0 and after 10 min (T10) of continuous stimulation. NADH values are expressed as a percent of maximum NADH values as described in Methods. *n* = 34 and 30 cells in old myocytes versus young myocytes, respectively. *P < 0.05 old versus young at same time point. †*P* < 0.05 T10 versus T0 within group. Values represent means ± SE

[NADH]_m_ levels in young and old myocytes are shown in Figure 3. There was a significant interaction between age and the effect of continuous contraction on [NADH]_m_. After 10 min of continuous stimulation, [NADH]_m_ levels significantly declined in old myocytes while [NADH]_m_ levels in young myocytes were maintained.

### Effects of rotenone on myocyte contractile properties

**Figure 4:**
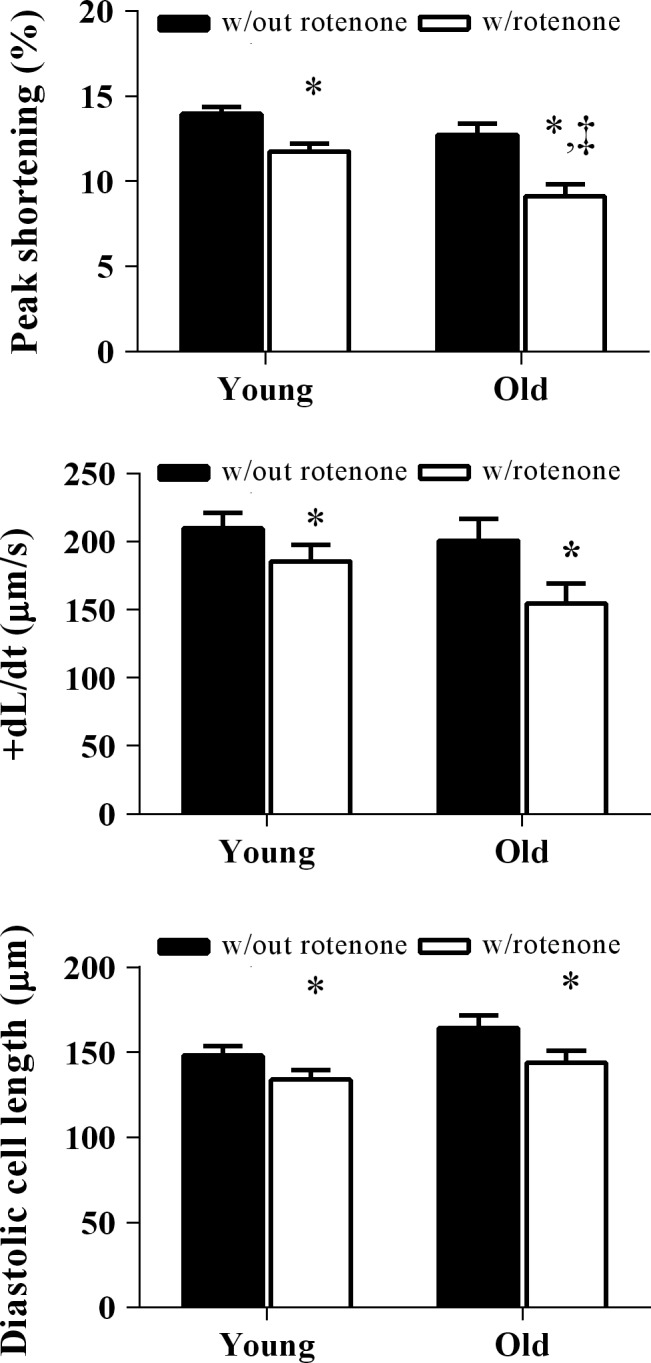
Rotenone was used to determine the effect of metabolic disruption on contractile performance. Contractile properties are expressed as absolute values before and after rotenone administration. Statistical analyses were performed on the change in contractile properties before and after rotenone administration (rotenone/without rotenone) between young and old myocytes. *n* = 28 cells and 26 cells in old myocytes versus young myocytes, respectively. *P < 0.05 main effect of rotenone treatment; ‡*P* < 0.05 between young and old myocytes. Values are means ± SE

In order to further determine the role of metabolic changes on cell shortening properties, we disrupted mitochondrial function in stimulated cells using rotenone. Rotenone is an irreversible inhibitor of complex I in the electron transport chain, and we assayed the impact that this metabolic stressor had on contractile function in old and young myocytes. There was a main effect of age and rotenone treatment on peak shortening in myocytes (*P* < 0.05). Although peak shortening declined in both young and old myocytes, rotenone had a greater effect (*P* < 0.05) on the decline in peak shortening in old myocytes compared to young (old = 9.1 ± 0.7 vs. young = 11.8 ± 0.4) when stimulated at 2 Hz (Fig. 4). A main effect of rotenone treatment was determined for the decline in +dL/dt (Fig. 4) in young and old myocytes, with no differences in +dL/dt in either groups respective of treatment. Diastolic cell length also declined (*P* < 0.05) as a result of rotenone treatment in both young and old cardiac myocytes, with no age‐related differences observed.

### Isoproteronol effects on myocyte shortening properties

**Figure 5:**
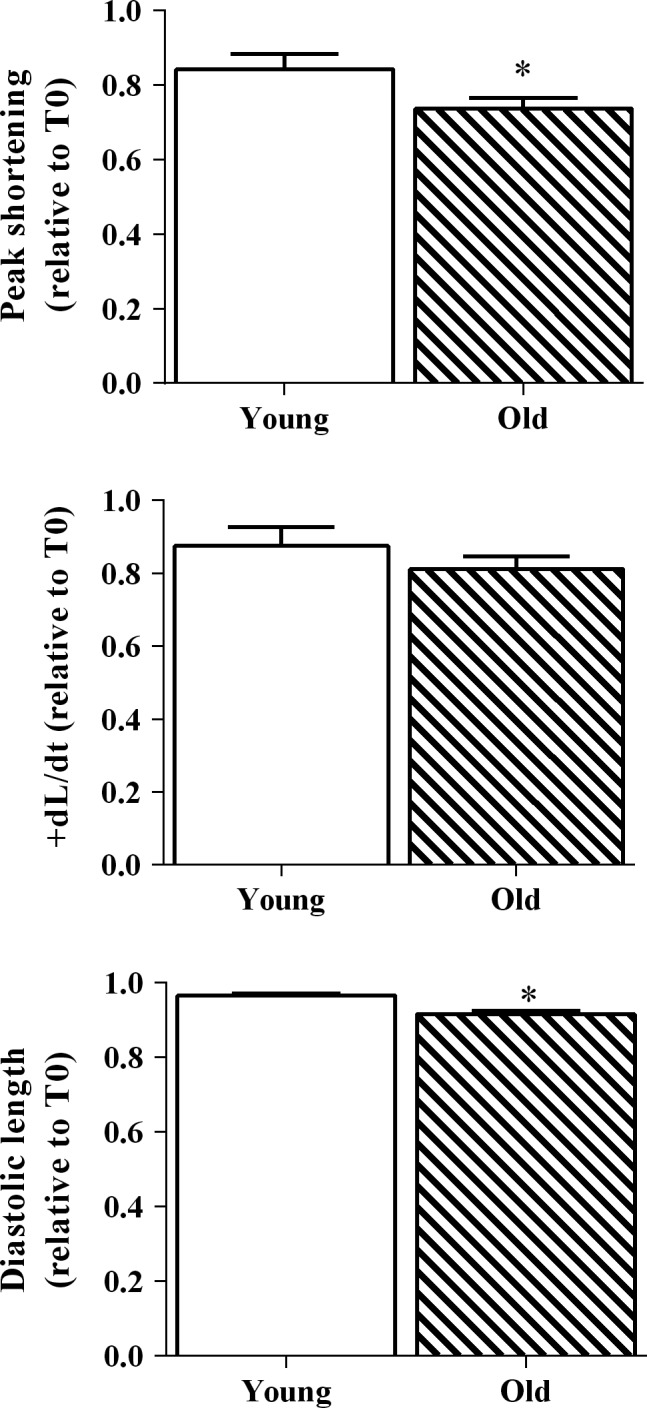
Isoproterenol was administered to increase cellular stress on young (open bars) and old myocytes (slashed bars). Contractile properties are expressed as a ratio of the value after 5 min of isoproterenol exposure and the initial value (T5/T0). *n* = 40 cells in old myocytes and young myocytes, respectively *P < 0.05 old versus young. Values indicate means ± SE

Isoproteronol (ISO) is a nonselective *β*‐adrenergic agonist, which is known to augment the work output of cardiac myocytes. We administered 100 nmol/L ISO to young and old cardiac myocytes throughout the duration of 5 min of continuous electrical stimulation in order to increase the contractile workload. Old myocytes demonstrated greater declines in peak shortening and diastolic cell length (*P* < 0.05) over time in the presence of ISO compared to young myocytes (Fig. 5). The decline in the +dL/dt over time was not different between young and old myocytes.

## Discussion

The primary findings of this study were that [NADH]_m_ declined in old myocytes during 10 min of continuous stimulation at 4 Hz (Fig. 3), while this stimulation protocol had no effect on NADH in young myocytes. To our knowledge, this is the first report of an age‐related difference in the ability of cells to maintain appropriate NADH levels. We also show that this change in metabolic function may also impact the cells contractile function. Although there was no significant difference in the contractile function decline over 10 min of continuous stimulation at 4 Hz between young and old myocytes, we did find that when the cells were under increased metabolic or workload stress, there was an age‐dependent decline in contractile performance in old compared to young myocytes (Figs. 4, 5), which is consistent with a previous report demonstrating the susceptibility of aged hearts to metabolic (Sudhir et al. 2011) and workload stress (Fleg et al. 1995). These results suggest that diminished metabolic reserve may play a role in stress‐induced contractile dysfunction in aged myocytes.

In this study, we assessed for changes in contractile function over time by measuring contractile properties at two different time points during continuous stimulation. We hypothesized that any observed differences in contractile performance over time would be a reflection of cellular energetic differences between young and old myocytes. Notably, we found that while myocyte contractile function did decline over time, the observed decline was reversible and readily recovers (Fig. 2). To us, this suggests that these functional changes are related to metabolic changes within the cell.

To explore the relationship between contractile function and metabolic capacity, we utilized steady‐state NADH autofluorescence measurements to assay mitochondrial function in single cardiac myocytes. The fluorescence of single cells has been previously used as a measure of mitochondrial NADH (White and Wittenberg 1993; Griffiths et al. 1998; Brandes and Bers 2002; Heinzel et al. 2006; Jo et al. 2006). Although single‐cell fluorescence cannot distinguish between cytosolic and mitochondrial NADH, over 80% of a cells autofluorescence in myocytes has been shown to originate from mitochondrial NADH (Eng et al. 1989; Jo et al. 2006). Previous NADH measures in single myocytes focused on changes in NADH with acute changes in workload (Griffiths et al. 1997, 1998; Heinzel et al. 2006; Jo et al. 2006). These experiments demonstrated that with an increase in workload, NADH fluorescence initially decreased but then slowly recovered to a new, higher, steady state (Brandes and Bers 2002; Jo et al. 2006). Our results demonstrated that steady‐state [NADH]_m_ levels declined in old myocytes over 10 min of continuous stimulation, whereas young myocytes maintained steady‐state [NADH]_m_ levels over that same duration. Although most previous studies have examined acute changes in NADH, one study demonstrated that, in young healthy myocytes, steady‐state [NADH]_m_ levels are maintained at a given workload for up to 30 min (Jo et al. 2006). We also observed a maintenance of [NADH]m levels in young myocytes but not old, suggesting that the decline in steady‐state [NADH]_m_ levels in old myocytes reflects altered mitochondrial function with age.

Baseline contractile performance in our study was not different between old and young myocytes at either T0 or T10, suggesting there were no age‐related alterations in fundamental properties of contraction. The influence of age on contractile function in isolated myocytes has been studied previously. Some studies demonstrated that aged myocytes have increased time to peak shortening (Fraticelli et al. 1989; Sakai et al. 1992), decreased velocity of sarcomere shortening (Wahr et al. 2000), and a greater decrement in peak shortening with increases in stimulation frequency (i.e., workload) (Li et al. 2005). In contrast, other studies demonstrated no age‐related differences in contractile function (peak shortening and rate of shortening) (Fraticelli et al. 1989; Sakai et al. 1992; Li et al. 2005). Some of these discrepancies may reflect differences in experimental design and conditions including temperature, stimulation frequency, and the contractile property being studied. We chose to stimulate the cells at 37°C using a frequency of 4 Hz which closely mimics the in vivo resting heart rate in the FBN strain (Hacker et al. 2006). Some previous studies using intact aging myocytes have used subphysiological stimulation frequencies and subphysiologic temperature (Fraticelli et al. 1989; Li et al. 2005). It was surprising that under baseline conditions, while NADH levels decreased over time in old myocytes, there was not a corresponding decrease in contractile function in the old myocytes over this time. It could be that this stimulation protocol (4 Hz), while sufficient to elicit a decrease in NADH levels, was not sufficiently stressful to unmask age‐related contractile dysfunction. This possibility is supported by the fact that, when put under severe stress (isoproterenol or rotenone) there was a clear decline in contractile function in the old myocytes.

Despite the lack of age‐related changes in baseline contractile properties, our data indicate that when the cell is put under metabolic or workload stress, contractile performance declines over time to a greater extent in aged myocytes compared to young cells. Rotenone is known to be a high‐affinity, irreversible inhibitor of complex I of the electron transport chain. In one study, rotenone administration to an isolated buffer‐perfused rabbit heart resulted in a developed pressure decline to 60% of control values (Lesnefsky et al. 2004). In our study, old myocytes demonstrated increased sensitivity to rotenone treatment compared to young myocytes, suggesting that contractile function is more sensitive to metabolic disruption in the older myocytes. When coupled with our observation that NADH levels were not maintained in the old myocytes during continuous pacing, the general overall mitochondrial performance in the old myocytes appears to be diminished.

Isoproterenol is a nonselective *β*‐adrenergic agonist acting through cAMP signaling leading to increases in protein phosphorylation of cardiac TnI (Jiang et al. 1993), phospholamban (Jiang et al. 1993), myosin binding protein C (Jiang et al. 1993), ryanodine receptor (RyR2) (Poláková et al. 2015), and L‐type Ca^2+^ channel (Reuter et al. 1982; Minobe et al. 2014). The effects of isoproterenol on contractility include increases in peak shortening/tension (Belardinelli and Isenberg 1983; Harding et al. 1988; Jiang et al. 1993; Xiao and Lakatta 1993), rate of shortening/force development (Belardinelli and Isenberg 1983; Harding et al. 1988; Jiang et al. 1993; Xiao and Lakatta 1993), and rate of relaxation (Harding et al. 1988; Jiang et al. 1993; Xiao and Lakatta 1993). The net effect of these changes is to increase the work output of the cell. We took advantage of these changes to study contractile performance over time with increasing workload. Aged myocytes in the presence of isoproterenol showed an augmented decline in contractile performance over time compared to young. Reduced myocardial contractile performance during *β*‐adrenergic agonist stimulation has been determined in aged hearts (Sakai et al. 1992; Jiang et al. 1993; Xiao et al. 1998). However, this is the first study to our knowledge to examine differences in contractile performance over time during continuous stimulation with isoproterenol. We assayed cellular contractile function over time (T5/T0), therefore, any age‐related difference in baseline *β*‐adrenergic sensitivity are normalized away. Taken together, we found a greater contractile functional decline in old versus young myocytes both when rotenone increased metabolic stress and when isoproterenol induced a workload stress on myocytes. These results suggest that metabolic processes may be unable to keep up with the increased demand in aged myocytes.

### Mechanisms and consequences of altered energetics and mitochondrial function with age

Mitochondrial functional changes have been observed in the aging heart, with changes ranging from altered substrate metabolism (Abu‐Erreish et al. 1977; McMillin et al. 1993), decreased ETC enzyme activity (Paradies et al. 1997; Kumaran et al. 2005; Preston et al. 2008; Gómez et al. 2009), reduced mitochondrial respiration (Preston et al. 2008; Hunter et al. 2012) to mitochondrial DNA deletions and enzymatic abnormalities (Wanagat et al. 2002). Mitochondrial dysfunction has been demonstrated in parallel with age and pressure‐overload hypertrophy (Wanagat et al. 2002; Doenst et al. 2010), but our study is the first to our knowledge to demonstrate the relationship between mitochondrial function (as measured by [NADH]_m_) and contractile function by simultaneously measuring both parameters in aged cardiac myocytes. Although we found no contractile functional differences during our baseline assay (4 Hz), we did observe a reduction in [NADH]_m_ in aged myocytes over 10 min of stimulation. What is clear from our findings is when either a workload or metabolic stress is induced, old myocytes demonstrate a greater decline in contractile function as compared to young. The inability of the older myocyte to maintain steady‐state NADH levels compared to the young myocyte suggests a diminished mitochondrial functional capacity which might place the myocyte at increased risk of energy depletion during acute increases in demand. Our results are supported by previous studies demonstrating age‐related reductions in the enzyme activity of Krebs cycle dehydrogenases (isocitrate, malate, and *α*‐ketoglutarate) in rat myocardium (Kumaran et al. 2005). In addition, studies have shown that the slow recovery of NADH to steady‐state levels is due to an upregulation of NADH formation through the increase in mitochondrial [Ca^2+^] (Brandes and Bers 2002; Jo et al. 2006). Although we did not measure mitochondrial Ca^2+^ levels, Ca^2+^ uptake into the mitochondria is important for the activation of several mitochondrial dehydrogenases, particularly pyruvate dehydrogenase (PDH), isocitrate dehydrogenase, and *α*‐ketoglutarate dehydrogenase (O'Donnell et al. 1998). However, little is known about the effect of age on mitochondrial Ca^2+^ uptake, thus more research is needed in this area. Although we believe that the assay used in this study to determine changes in contractile function over time as a method to assess the contractile consequences of age‐related metabolic deficits, we cannot rule out the possibility that stress‐induced contractile dysfunction found in old myocytes herein may be in part be due to other age‐related changes in the microenvironment such as proteins that span the sarcomere, intercalated disk, and the sarcolemma which are modified by age (Sessions and Engler 2016).

## Conclusion

Although cardiac myocyte contractile function did not show any age‐dependent differences under resting conditions, old myocytes demonstrate impaired contractile function to a workload (ISO) and metabolic (rotenone) stress compared to young myocytes. Even under a stimulation frequency closely resembling resting heart rate, old myocytes exhibit decrements in [NADH]_m_ over time. These data suggest that older cardiac myocytes are less able to maintain energetic homeostasis at rest and have a lower mitochondrial capacity to respond to increased demand. These findings indicate that cellular energy metabolism is compromised during senescence and may play a role in the decreasing cardiac reserve of the older heart.

## Conflict of Interest

None declared.
